# Nox2 contributes to the arterial endothelial specification of mouse induced pluripotent stem cells by upregulating Notch signaling

**DOI:** 10.1038/srep33737

**Published:** 2016-09-19

**Authors:** Xueling Kang, Xiangxiang Wei, Xinhong Wang, Li Jiang, Cong Niu, Jianyi Zhang, Sifeng Chen, Dan Meng

**Affiliations:** 1 Department of Physiology and Pathophysiology, School of Basic Medical Sciences, Fudan University, Shanghai 200032, China; 2 Division of Cardiology, Department of Medicine, Stem Cell Institute, University of Minnesota Medical School, Minneapolis, MN 55455, USA

## Abstract

Reactive oxygen species (ROS) have a crucial role in stem-cell differentiation; however, the mechanisms by which ROS regulate the differentiation of stem cells into endothelial cells (ECs) are unknown. Here, we determine the role of ROS produced by NADPH oxidase 2 (Nox2) in the endothelial-lineage specification of mouse induced-pluripotent stem cells (miPSCs). When wild-type (WT) and Nox2-knockout (Nox2^−/−^) miPSCs were differentiated into ECs (miPSC-ECs), the expression of endothelial markers, arterial endothelial markers, pro-angiogenic cytokines, and Notch pathway components was suppressed in the Nox2^−/−^ cells but increased in both WT and Nox2^−/−^ miPSCs when Nox2 expression was upregulated. Higher levels of Nox2 expression increased Notch signaling and arterial EC differentiation, and this increase was abolished by the inhibition of ROS generation or by the silencing of Notch1 expression. Nox2 deficiency was associated with declines in the survival and angiogenic potency of miPSC-ECs, and capillary and arterial density were lower in the ischemic limbs of mice after treatment with Nox2^−/−^ miPSC-ECs than WT miPSC-EC treatment. Taken together, these observations indicate that Nox2-mediated ROS production promotes arterial EC specification in differentiating miPSCs by activating the Notch signaling pathway and contributes to the angiogenic potency of transplanted miPSC-derived ECs.

Endothelial cells (ECs) generated from induced pluripotent stem cells (iPSCs) are among the most promising therapeutics in vascular medicine; however, they may be even more effective when matched to the type of tissue that is in need of repair^1^,^2^. Thus, methods for directing the differentiation of iPSCs into a specific EC subtype, such as arterial or venous ECs^3^,^4^, may enhance the effectiveness of cardiovascular cell therapy. The molecular mechanisms responsible for EC specification have yet to be thoroughly characterized but could include the Notch signaling pathway, which is known to be important for regulating arterial-venous cell specification^5^,^6^.

In ECs, Notch signaling is activated when Notch1 or Notch4 binds any of several Notch ligands, including Delta-like (Dll) 1, Dll4, Jagged1, and Jagged2, which are expressed in arteries but not in veins^7^,^8^. Notch signaling is mediated by the Notch intracellular domain (NICD) and the transcription factor RBP-J, and studies in animals have shown that Notch1, Notch4, RBP-J, and Dll1, as well as two downstream targets of Notch, Hes1 and Hey1, are essential for arterial formation in the developing vasculature^9^,^10^. Notch signaling may also regulate arterial EC specification in response to canonical Wnt signaling and the upstream activity of vascular endothelial growth factor (VEGF)^11^,^12^.

Reactive oxygen species (ROS) such as hydrogen peroxide and superoxide, as well as the balance between ROS generation and elimination (i.e., the cell’s “redox status”) are important regulators of cell survival and proliferation^13^,^14^,^15^. In stem cells, ROS influence interactions between the cells and their local microenvironment^16^, contribute to the maintenance of “stemness,” and participate in stem-cell differentiation^17^,^18^; for example, we have previously shown that hydrogen peroxide upregulates osteoblast- and adipocyte-associated gene expression in differentiating mouse iPSCs (miPSCs)^19^, and another recent study has reported that the accumulation of ROS under low-oxygen conditions promotes the differentiation of human pluripotent stem cells into vascular ECs^1^. A substantial amount of cellular ROS production occurs through the activity of NADPH oxidases (NOX)^18^,^20^, including Nox2, which is highly expressed in stem and progenitor cells^20^. Nox2 expression occurs in embryonic stem cells (ESCs) from an early stage of development and is synchronized with changes in the expression of other subunits of NADPH oxidases, such as p22phox, p47phox, and p67phox, which suggests that Nox2 participates in ESC differentiation^21^. Nox2-mediated ROS production has also been linked to the differentiation of cardiac precursor cells into smooth- and cardiac-muscle cells^22^, to progenitor-cell expansion, and to the mobilization of bone-marrow progenitor cells in response to ischemic injury^23^.

Only a few studies have identified a potential link between cellular ROS production and EC-fate determination in stem/progenitor cells^24^,^25^; however, the results presented here indicate that Nox2 gene expression is ~10-fold greater in ECs that have been differentiated from miPSCs (miPSC-ECs) than in the miPSCs themselves. Thus, we generated miPSCs from wild-type (WT) and Nox2-knockout (WT miPSCs and Nox2^−/−^ miPSCs, respectively) mouse embryonic fibroblasts (MEFs), differentiated the WT miPSCs and Nox2^−/−^ miPSCs into ECs (WT miPSC-ECs and Nox2^−/−^ miPSC-ECs, respectively), and then evaluated the WT miPSC-ECs and Nox2^−/−^ miPSC-ECs in a series of *in-vitro* experiments, as well as *in-vivo* models of angiogenesis (Matrigel-plug) and peripheral ischemia (murine hind-limb ischemia). Our results provide the first evidence that Nox2-mediated ROS production activates the Notch-signaling pathway in differentiating miPSCs, and that this mechanism has a key role in endothelial-lineage specification and in the angiogenic potency of miPSC-ECs.

Detailed Expanded Methods are available in the Supplementary Information.

## Mice

The Nox2^−/−^ mice (Stock Number 002365) were purchased from Jackson Laboratories (Bar Harbor, ME). C57BL/6J (Stock Number J000664) and nonobese diabetic (NOD)/severe combined immunodeficiency (SCID) mice (Stock Number T001521) were purchased from the Model Animal Research Center of Nanjing University (Nanjing, China). All experiments were reviewed and approved by the Ethics Committee of Experimental Research, Fudan University Shanghai Medical College and were consistent with the “Guide for the Care and Use of Laboratory Animals” published by the National Institutes of Health (NIH) of the United States.

## Cell culture

Mouse embryonic fibroblasts (MEFs) were isolated from embryos on embryonic day 13.5 and cultured in Dulbecco’s modified Eagle medium with 10% fetal bovine serum^26^. Mouse iPSCs were grown on mitomycin C-treated MEF feeders in standard ESC medium (Dulbecco’s modified Eagle medium supplemented with 2 mM l-glutamine, 0.1 mM nonessential amino acids, 1 mM sodium pyruvate, 0.1 mM β-mercaptoethanol, 50 U/mL penicillin, 50 μg/mL streptomycin, and 0.1 μg/mL leukemia inhibitory factor) with 10% knockout serum replacement. All culture reagents were from Invitrogen (Carlsbad, CA) unless otherwise indicated.

## Viral transduction of fibroblasts and miPSC induction

Mouse iPSCs were generated as described by Takahashi *et al*.^26^. Briefly, MEFs were reprogrammed with lentiviral vectors that expressed the mouse transcription factors Oct4 (O), Klf2 (K), Sox2 (S) and c-Myc (M). Doxycyline-inducible lentivirus expressing mouse transcription factor of OKSM were used to transduce the fibroblasts. The plasmid (FUW-OSKM, #20308) was purchased from Addgene (Cambridge, MA). The lentiviruses were packed in 293T cells and concentrated with PEG-it™ virus precipitation solution (System Biosciences, Mountain View, CA). Transduction was induced with polybrene (10 μg/mL), and the cells were transduced twice, 24 hours apart. Two days after the second transduction, the transduction medium was replaced with ESC medium, and the ESC medium was changed daily until miPSC colonies were selected on Day 12–14 after transduction. The selected miPSCs were expanded in culture with feeder cells.

## miPSC-EC differentiation and purification

miPSC-EC differentiation was performed as previously described^27^. miPSCs were cultured on ultra-low attachment plates (Corning Glass Works, Corning, NY) in differentiation media (Dulbecco’s modified Eagle medium containing 20% knockout serum replacement, 0.05 mmol/L β-mercaptoethanol, 1% non-essential amino acids, and 50 ng/mL BMP-4). Four days later, the miPSCs had grown to form embryoid bodies, which were collected, seeded onto 0.2% gelatin-coated dishes and cultured in differentiation media with VEGF (50 ng/mL), but without BMP-4, for 10 days. The media was changed every two days, and 10 μM SB431542 was added three days later and maintained for 7 days. After differentiation, the miPSC-ECs were dissociated with accutase, washed with 1× phosphate-buffered saline (PBS) containing 5% bovine serum albumin (BSA), passed through a 70-μm cell strainer (BD Biosciences, Bedford, MA), labeled with magnetic beads conjugated to CD31 antibodies, and purified via Magnetic Activated Cell Sorting (MACS; Miltenyi Biotech, Bergisch Gladbach, Germany). The purified miPSC-ECs were identified by flow-cytometry analysis of CD31 expression with over 95% purity and were expanded in ECM-2 media. The miPSC-ECs were characterized via the expression of CD31, CD144, and vWF.

## Flow Cytometry Analysis

Flow-cytometry analysis of CD31 or Flk-1 expression was performed with a FACSCalibur™ flow cytometer (BD Biosciences, San Jose, CA). Isotype control matching the immunoglobulin subtype was stained analogously to reveal non-specific binding. The data were analyzed with CellQuest software (BD Biosciences, San Jose, CA).

## Quantitative real time reverse transcription-polymerase chain reaction (RT-PCR)

RT-PCR analyses were performed as described previously^28^. Total RNA was extracted with TRIzol reagent (Invitrogen, Carlsbad, CA) as directed by the manufacturer’s instructions, cDNA was synthesized with SuperScript Reverse Transcriptase (Fermentas, Glen Burnie, MD), and the RT-PCR analysis was performed with SYBR Green PCR master mix (Toyobo, Osaka, Japan). Samples were analyzed on a Bio-Rad real-time analyzer (Bio-Rad Laboratories, Hercules, CA), and measurements were normalized to glyceraldehyde-3-phosphate dehydrogenase (GADPH) mRNA levels, and a control sample (calibrator set to 1) was used to calculate the relative values. Primer sequences are listed in Supplementary Table 1.

## Immunoblot analysis

Immunoblot analysis was performed as described previously^29^,^30^. Antibodies to CD31, CD144, VEGF, Angiopoietin 1, EphrinB2, ALK1, Jagged 1, Dll4, Cleaved Notch1, Hes1, Hey1 and β-actin were used.

## Measurement of Intracellular ROS Levels

ROS production was monitored via the DCF-DA and dihydroethidium (DHE) assays as described previously^28^,^30^. Briefly, the cells were incubated with DCF-DA (5 μM) for 15 min or DHE (5 μM) for 20 min in a light-protected humidified chamber at 37 °C and then harvested and resuspended in PBS. DCF fluorescence was excited at 488 nm and monitored at 540 nm, and DHE fluorescent was excited at 535 nm and monitored at 610 nm. Fluorescence levels were measured with a flow cytometer (BD Biosciences, San Jose, CA). Each experiment was performed three times in three replicate wells.

## *In-vivo* Matrigel plug model

The purified miPSC-ECs were expanded in ECM-2 for 7 days; then, 1 × 10^6^ cells were mixed with Matrigel to a final volume of 500 μL, and the mixture was subcutaneously injected into the mid-lower abdominal region of immunodeficient male NOD/SCID mice (6 weeks). Seven days later, the animals were euthanized with sodium pentobarbital (50 mg/kg i.p.), and the plugs were excised, paraffin-embedded, and cut into 5-μm sections. Capillary density was evaluated in sections stained for the expression of CD31, an EC-specific marker. Capillaries were identified by positive staining for CD31. Sections were viewed at 20x magnification, and vessels were counted in 10 high-power fields in each of the four tissue sections per animal. For analyses of hemoglobin content, the excised plug was homogenized in 100 μL PBS and centrifuged; then, the supernatant was evaluated via the Drabkin assay. Hemoglobin content was calculated by comparing the results for each sample to a standard curve generated from measurements of stock solutions containing known quantities of hemoglobin.

## Murine hind-limb ischemia model

Unilateral hind-limb ischemia (HLI) was induced in eight-week-old male C57BL/6J mice as described previously^31^. Briefly, the animals were anesthetized with sodium pentobarbital (50 mg/kg i.p.), and the right femoral artery was exposed under a dissection microscope; then, the proximal region of the femoral artery and the distal portion of the saphenous artery were ligated, and the artery was excised. Animals were randomly assigned to treatment with WT miPSC-ECs, Nox2^−/−^ miPSC-ECs, or saline, and the treatments were delivered immediately after HLI injury. The purified miPSC-ECs had been transfected with adenoviruses coding for eGFP expression before administration, and 1 × 10^6^ cells were administered to each animal in the cell-treatment groups. The cells were suspended in 100 μL saline and delivered via intramuscular injection to 2 sites in the adductor muscle and 2 sites in the gastrocnemius muscle. Blood flow measurements were performed with a MoorLDI2–2λ laser Doppler imaging system (Moor Instruments, Devon, UK). Mice were anesthetized and positioned on a heating plate at (37 °C) to minimize temperature variation, and measurements in the ischemic limb were normalized to measurements in the uninjured contralateral limb. Mice were sacrificed on day 7 or 14 after injury and treatment; then, the adductor muscles were harvested, formaldehyde fixed, paraffin embedded, sectioned, and stained with CD31 antibody. Transplanted cells were identified via eGFP fluorescence. Capillaries in tissue sections were stained using a CD31 antibody and arterioles were visualized using an α-smooth muscle actin (SMA) antibody. Capillary or arteriole density was assessed by counting the number of capillaries or arterioles in 10 high-powered fields in each of the four tissue sections and then expressing the data as capillaries/mm^2^ or arterioles/mm^2^.

## Statistical analysis

Data are expressed as mean ± SEM. Differences among three or more groups were evaluated for significance via one-way analysis of variance and the Bonferroni post-hoc test; differences between two groups were evaluated via the two-tailed student *t*-test. A *P* value of less than 0.05 was considered significant.

## Results

### Generation and characterization of Nox2^−/−^ miPSCs

**Figure 1:**
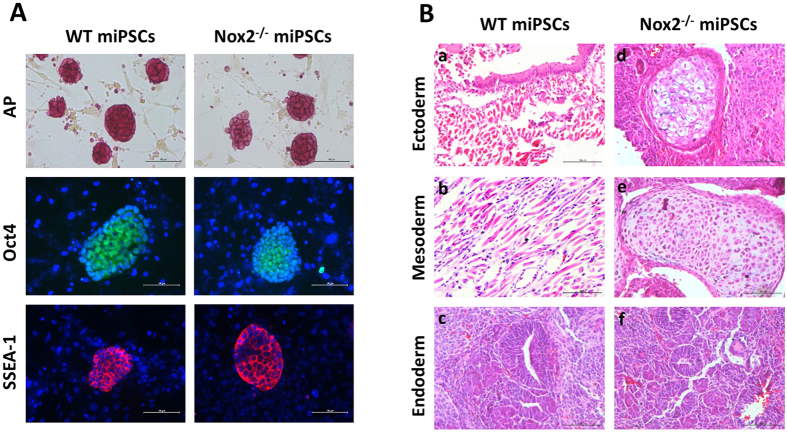
Generation and characterization of Nox2^−/−^ miPSCs. (**A**) WT miPSCs and Nox2^−/−^ miPSCs possessed alkaline phosphatase activity, and were positive for both Oct-4 and SSEA-1 by immunofluorescence staining. Scale bar: 100 μm. (**B**) Histological analysis of teratomas formed from grafted colonies of WT miPSCs (a–c) and Nox2^−/−^ miPSCs (d–f) in adult SCID mice. The tissues were stained with hematoxylin and eosin to document cells originating from the three germ layers: squamous epithelium (a) and sebaceous gland (d) (ectoderm); muscle (b) and cartilage (e) (mesoderm); endocrine gland (c,f) (endoderm). Scale bar: 100 μm.

miPSCs were generated by transfecting MEFs with lentiviruses coding for the Oct4, Sox2, c-Myc, and Klf4 transcription factors; miPSCs with normal levels of Nox2 expression (WT miPSCs) were generated from WT MEFs, and miPSCs lacking Nox2 expression (Nox2^−/−^ miPSCs) were generated from Nox2^−/−^ MEFs. Nox2^−/−^ and WT miPSCs were morphologically similar; both expressed Oct4 and SSEA-1 (Fig. 1A), were positive for alkaline-phosphatase (AP) activity, and produced teratomas containing cells from all three developmental germ layers (Fig. 1B). WT miPSCs expressed substantial amounts of Nox2 and Nox4 (Supplementary Figure 1A), while Nox1 expression was observed at low levels, and Nox3 and Nox5 were undetectable. mRNA measurements in Nox2^−/−^ miPSCs confirmed that Nox2 expression had been knocked out, and that the expression of Nox1, Nox4, p22phox, p47phox, and p67phox in Nox2^−/−^ miPSCs and WT miPSCs was similar (Supplementary Figure 1B). However, the loss of Nox2 expression led to significant declines in measurements of NADPH oxidase activity (Supplementary Figure 1C) and cellular ROS production (Supplementary Figure 1D,E).

### Nox2-derived ROS contributes to the differentiation of miPSCs into miPSC-ECs

**Figure 2:**
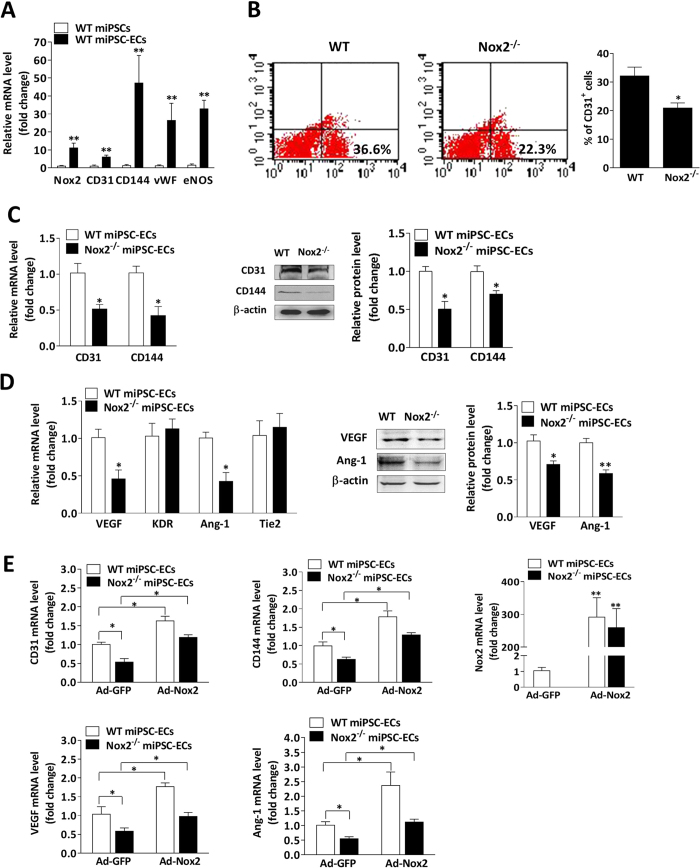
The loss of Nox2 expression suppresses the differentiation of miPSCs into miPSC-ECs. (**A**) Real-time PCR shows Nox2 parallel expression with EC markers at the mRNA levels in miPSC-ECs at day 14 of differentiation (n = 3; **P < 0.01 vs. WT miPSCs). (**B**) Flow cytometry analysis of ECs derived from WT miPSCs and Nox2^−/−^ miPSCs using specific antibodies to CD31 at day 14 of differentiation (n = 3; *P < 0.05 vs. WT miPSC). (**C**) The purified WT miPSC-ECs and Nox2^−/−^ miPSC-ECs were measured for the expression of endothelial markers, showing the decrease of CD31 and CD144 mRNA levels in Nox2^−/−^ miPSC-ECs (left panel). The reduced expression of CD31 and CD144 in Nox2^−/−^ miPSC-ECs was further confirmed by immunoblotting (right panel, n = 3; *P < 0.05 vs. WT miPSC-ECs). (**D**) Quantitative PCR analyses and immunobloting show the decrease of VEGF and angiopoietin-1 mRNA and protein levels in Nox2^−/−^ miPSC-ECs (n = 3; *P < 0.05, **P < 0.01 vs. WT miPSC-ECs). (**E**) Nox2 overexpression using Ad-Nox2 in WT miPSC-ECs and Nox2^−/−^ miPSC-ECs, showing the increase in CD31, CD144, VEGF and Ang-1 expression at mRNA levels (n = 3; *P < 0.05, **P < 0.01).

Nox2 and Nox4 expression gradually increased in both WT miPSCs (Supplementary Figure 2A,C) and in mouse embryonic stem cells (mESCs) (Supplementary Figure 2B) as the cells were differentiated into ECs, and mRNA levels for markers of EC identity (CD31, CD144, von Willebrand factor [vWf], and endothelial nitric oxide synthase [eNOS]) were significantly greater in WT miPSC-ECs than in WT miPSCs on day 14 after differentiation was initiated (Fig. 2A). Flow cytometry analysis indicated that after 5 days of differentiation, cells expressing the vascular-progenitor-cell marker Flk-1 were less common in differentiating Nox2^−/−^ miPSCs than in WT miPSCs (Supplementary Figure 3A), while the number of cells that expressed CD31 (Fig. 2B), as well as the mRNA and protein levels of both CD31 and CD144, were significantly lower in Nox2^−/−^ miPSC-ECs than in WT miPSC-ECs on day 14 (Fig. 2C). Notably, the expression of stemness markers (Oct4 and Nanog) in WT miPSCs and Nox2^−/−^ miPSCs declined at similar rates during the first six days of differentiation (Supplementary Figure 3B,C), which suggests that the impaired endothelial specification of Nox2^−/−^ miPSCs cannot be attributed to greater persistence of the undifferentiated state. Nox2 deficiency was also associated with declines in mRNA and/or protein levels of angiopoietin-1 (Ang-1) (Fig. 2D), platelet-derived growth factor (PDGF)-BB, fibroblast growth factor (FGF)-2 (Supplementary Figure 3D), hypoxia inducible factor-1α (HIF-1α) (Supplementary Figure 3E), and vascular endothelial growth factor (VEGF) (Fig. 2D; Supplementary Figure 3F) in miPSC-ECs, but not in the expression of VEGF receptor 2 (KDR), Ang-1 receptor 2 (Tie-2), or angiopoietin-2 (Ang-2) (Supplementary Figure 3D). Furthermore, when the miPSC-ECs were transduced with adenoviruses coding for Nox2 (Ad-Nox2) or GFP (Ad-GFP), the Ad-Nox2 vector was associated with significantly higher mRNA levels of CD31, CD144, VEGF, and Ang1 in both WT and Nox2^−/−^ miPSC-ECs (Fig. 2E).

**Figure 3:**
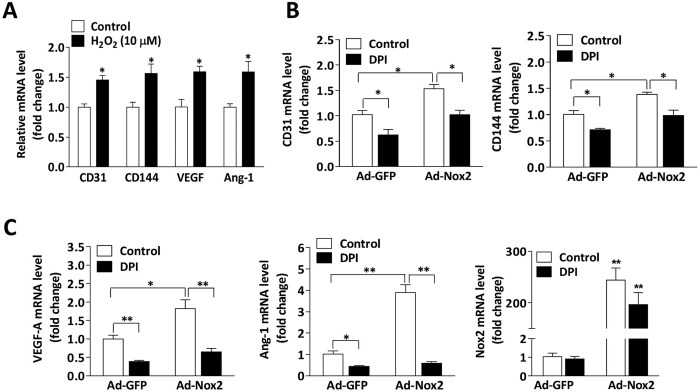
Nox2-derived ROS contributes to the differentiation of miPSCs into miPSC-ECs. (**A**) Exogenous H_2_O_2_ (10 μM) was administered at day 2, 3 and 4, and quantitative PCR analyses showed the increase of endothelial markers CD31, CD144, VEGF and Ang-1 mRNA levels in WT miPSC at day 5 of differentiation in response to H_2_O_2_ stimulation (n = 3; *P < 0.05 vs. control). (**B**,**C**) Nox2 overexpression using Ad-Nox2 in WT miPSCs during EC differentiation at day 2, and cells were treated with or without DPI (1 μM) at day 4 for 48 hours. Quantitative PCR analyses showing the increase of CD31 and CD144 (**B**), as well as VEGF and Ang-1 (**C**) mRNA levels induced by Nox2 overexpression was suppressed by DPI. (n = 3; *P < 0.05, **P < 0.01).

Nox2 deficiency was also associated with declines in endothelial-marker expression after just five days of differentiation (data not shown), so we investigated the effect of exogenous ROS on the expression of endothelial markers and angiogenic proteins in WT miPSCs at an earlier stage of endothelial differentiation (day 5) by exposing the cells to low levels of hydrogen peroxide (H_2_O_2_) or to diphenylene iodonium (DPI), which inhibits ROS production. mRNA measurements of CD31, CD144, VEGF, and Ang-1 expression increased significantly when WT miPSCs were cultured in the presence of 10 μM H_2_O_2_ (Fig. 3A) and declined significantly when Ad-GFP– or Ad-Nox2–transfected miPSCs were cultured with DPI (Fig. 3B,C). Collectively, these observations suggest that Nox2 contributes to endothelial specification and to the expression of pro-angiogenic cytokines in differentiating miPSCs.

### Nox2 regulates arterial EC differentiation via a Notch-dependent pathway

**Figure 4:**
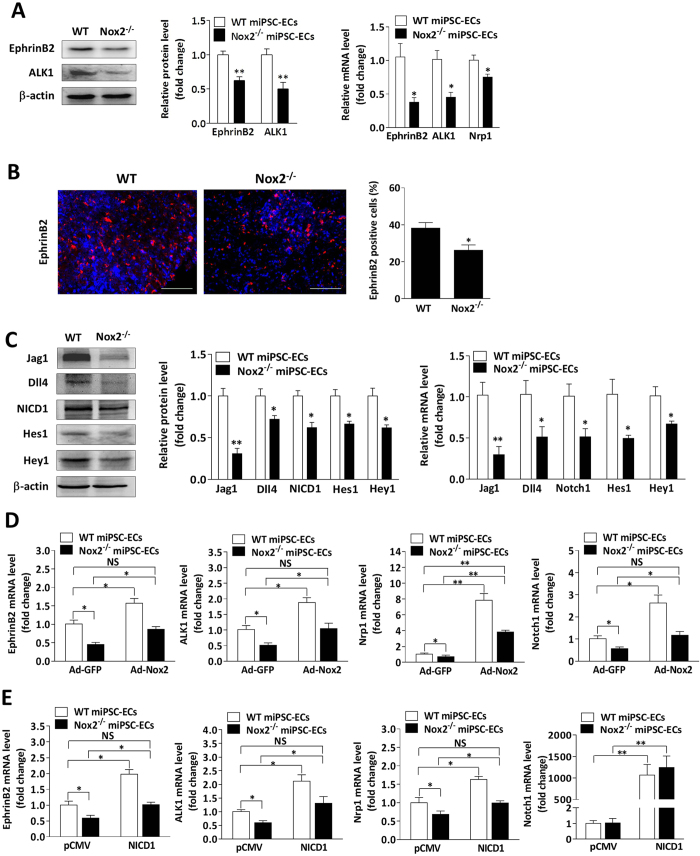
The loss of Nox2 expression suppresses arterial EC differentiation through a Notch-dependent pathway. (**A**) Immunobloting and quantitative PCR analyses show a suppression in arterial endothelial markers (EphrinB2, ALK1 and Nrp1) at mRNA and/or protein levels in Nox2^−/−^ miPSC-ECs (n = 3; *P < 0.05, **P < 0.01 vs. WT miPSC-ECs). (**B**) Immunohistochemical analysis of cells expressing EphrinB2 in 7-day-old embryoid bodies derived from WT miPSCs and Nox2^−/−^ miPSCs. The bar represents 100 μm. (n = 5; *P < 0.05 vs. WT miPSCs). (**C**) Quantitative PCR analyses and immunobloting show the decrease of Jagged 1, Dll4, Notch1, Hes1 and Hey1 at mRNA and/or protein levels in Nox2^−/−^ miPSC-ECs. (n = 3; *P < 0.05, **P < 0.01 vs. WT miPSC-ECs). (**D**) Nox2 overexpression using Ad-Nox2 in WT miPSC-ECs and Nox2^−/−^ miPSC-ECs, showing the increase of arterial EC makers and Notch1 at mRNA levels (n = 3; *P < 0.05, **P < 0.01). (**E**) Notch1 overexpression using a Notch1 constitutively active vector (NICD1) or transfection with the control vector (pCMV) in WT miPSC-ECs and Nox2^−/−^ miPSC-ECs, showing the increase of arterial EC makers and Notch1 at mRNA levels (n = 3; *P < 0.05, **P < 0.01).

**Figure 5:**
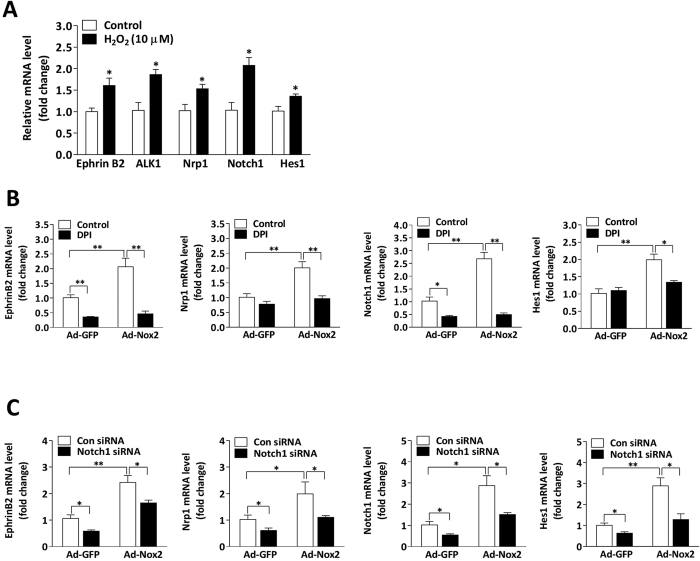
Nox2-derived ROS contributes to arterial EC differentiation of miPSCs. (**A**) Exogenous H_2_O_2_ (10 μM) was administered at day 2, 3 and 4, and quantitative PCR analyses showed the increase of arterial endothelial markers and Notch pathway components at mRNA levels in WT miPSC at day 5 of differentiation in response to H_2_O_2_ stimulation (n = 3; *P < 0.05 vs. control). (**B**,**C**) Nox2 overexpression using Ad-Nox2 or the control adenovirus (Ad-GFP) in WT miPSCs during EC differentiation at day 2, and cells were then treated with or without DPI (1 μM) at day 4 for 48 hours (**B**) or transfected with Notch1 siRNA or Control siRNA for 48 hours (**C**). mRNA levels of arterial endothelial markers and Notch pathway components were determined via real time RT-PCR at day 6. (n = 3; *P < 0.05, **P < 0.01).

The loss of Nox2 expression in miPSC-ECs was associated with significant declines in mRNA and/or protein levels of EphrinB2, activin receptor-like kinase 1 (ALK1), and neuropilin 1 (Nrp1) (Fig. 4A), all of which are expressed in arterial ECs. EphrinB2 (Fig. 4B), but not the venous marker EphB4 (data not shown), was also less frequently expressed by cells in embryoid bodies from Nox2^−/−^ miPSCs than in the cells of WT miPSC-derived embryoid bodies. Since EphrinB2 expression is induced by Notch signaling^12^, we investigated whether the expression of Notch ligands (Jagged 1 and Dll4), the Notch1 intracellular domain (NICD1), and Notch target genes (Hey1 and Hes1) was altered by the loss of Nox2 in miPSC-ECs. mRNA and/or protein levels of these Notch-pathway components were consistently lower in Nox2^−/−^ miPSC-ECs than in WT miPSC-ECs (Fig. 4C). However, the expression of arterial endothelial markers and Notch1 increased significantly when the cells were transfected with Ad-Nox2 (Fig. 4D) or when Notch activity was upregulated with a vector that coded for a constitutively active form of NICD1 (Fig. 4E). These genes expression also increased when the cells were cultured with low levels of H_2_O_2_ (Fig. 5A), and increased expression of genes induced by higher levels of Nox2 was abolished by the inhibition of ROS generation with DPI (Fig. 5B) or by the silencing of Notch1 expression (Fig. 5C). Collectively, these observations suggest that Nox2-mediated ROS production likely contributes to the differentiation of miPSCs into arterial ECs by stimulating the Notch signaling pathway.

### Nox2 contributes to the activity, survival, and angiogenic potency of miPSC-ECs

**Figure 6:**
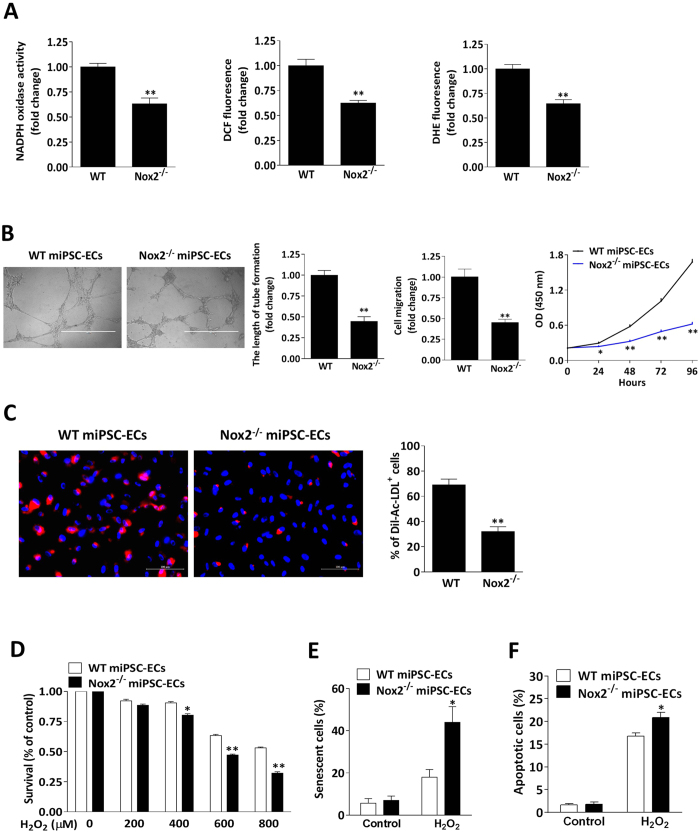
Characterization of Nox2^−/−^ miPSC-ECs. (**A**) The purified WT miPSC-ECs and Nox2^−/−^ miPSC-ECs were measured for NADPH oxidase activity and assessed for ROS production by DCF-DA or DHE method, showing the decrease of NADPH oxidase activity and ROS levels in Nox2^−/−^ miPSC-ECs (n = 3; **P < 0.01 vs. WT miPSC-ECs). (**B**) *In vitro* Matrigel assays of purified miPSC-ECs for tube formation. Scale bar, 1000 μm (left panel). WT miPSC-ECs and Nox2^−/−^ miPSC-ECs were allowed to migrate through a membrane by using transwell chambers for 10 hours, and migrated cells were counted (middle panel). Cell proliferation was analyzed at the indicated time points by BrdU incorporation method (right panel). Nox2^−/−^ miPSC-ECs displayed the reduced capacities for tube formation, cell migration and proliferation (n = 3; *P < 0.05, **P < 0.01 vs. WT miPSC-ECs). (**C**) Representative images and quantification show the reduction of Dil-Ac-LDL uptake in Nox2^−/−^ miPSC-ECs. Scale bar, 100 μm. (n = 4; **P < 0.01 vs. WT miPSC-ECs). (**D**) WT miPSC-ECs (white bars) and Nox2^−/−^ miPSC-ECs (black bars) were treated with the indicated concentrations of H_2_O_2_ for 24 hours, and cell viability was measured using CCK-8 assays. Nox2^−/−^ miPSC-ECs displayed the reduced viability in H_2_O_2_ concentrations of 400 μM or greater (n = 3; *P < 0.05, **P < 0.01 vs. WT miPSC-ECs). (**E**) WT and Nox2^−/−^ miPSC-ECs were treated with or without H_2_O_2_ (600 μM) for 6 hours. Cell senescence was measured using β-galactosidase staining. Nox2^−/−^ miPSC-ECs showed the increase of celluar senescence with H_2_O_2_ treatment (n = 3; *P < 0.05 vs. WT miPSC-ECs). (**F**) miPSC-ECs with or without 24 hours of H_2_O_2_ (600 μM) treatment were stained with annexin V-FITC and propidium iodide (PI), and analyzed by flow cytometry. The percentage of annexin V-positive cells (earlier apoptotic cells) of total cells is shown. Cellular apoptosis was significantly greater in Nox2^−/−^ miPSC-ECs (n = 3; *P < 0.05 vs. WT miPSC-ECs).

**Figure 7:**
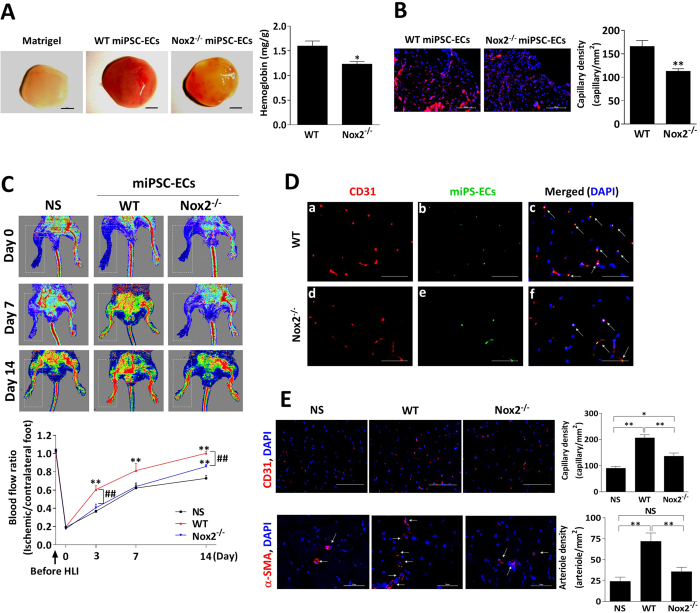
Nox2 deficiency reduces the capacity of miPSC-ECs for vascular repair in the ischemic limbs of mice. (**A**,**B**) Decreased angiogenesis in an *in-vivo* Matrigel plugs with Nox2^−/−^ miPSC-ECs. The purified WT miPSC-ECs or Nox2^−/−^ miPSC-ECs were mixed in Matrigel plugs and implanted subcutaneously into nude mice. After seven days, mice were euthanized and the Matrigel plugs were explanted. Representative micrographs of the Matrigel plugs. Scale bar: 2 mm (A, left panel). Perfusion of Matrigel plugs was determined by measuring the hemoglobin content (A, right panel). The Matrigel plugs were fixed, sectioned and stained for CD31 (for blood vessels). Scale bar: 200 μm (B, left panel). Quantitative analysis of capillary density in the Matrigel plugs is shown (B, right panel). (n = 6 per group; *P < 0.05, **P < 0.01 vs. WT miPSC-ECs). (**C**) C57BL/6 mice underwent hindlimb ischemia as described in Methods. The purified WT miPSC-ECs, Nox2^−/−^ miPSC-ECs, or saline (NS) was injected into the thigh adductor and gastrocnemius muscles. Images of laser doppler perfusion imaging at day 0, 7 and 14 after treatment (upper panels). Blood flow was measured with a laser Doppler imaging system, and recovery was quantified as the ratio of ischemic (right) leg to non-ischemic (left) leg. The perfusion ratio was greater in mice that received WT miPSC-ECs transplantation compared to those that received Nox2^−/−^ miPSC-ECs (lower panel, n = 8; **P < 0.01 vs. the NS-treated animals, ^##^*P* < 0.01 vs. Nox2^−/−^ miPSC-EC–treated animals). (**D**) Co-localization of miPSC-EC (GFP staining, green) with ECs (CD31 staining, red) in ischemic muscles 7 days after treatment. Nuclei were stained with DAPI (blue). Merged images were generated by overlay of DAPI, anti-CD31, and GFP staining (n = 5; Scale bar: 20 μm). (**E**) Immunofluorescent CD31 (upper panels; Scale bar: 100 μm) and αSMA (lower panels; Scale bar: 50 μm) staining of ischemic tissues from mice treated with saline or miPSC-ECs after 14 days. Both capillary density and arteriole density in the ischemic limbs were greater after treatment with WT miPSC-ECs than after Nox2^−/−^ miPSC-EC treatment (n = 6; *P < 0.05, **P < 0.01).

To determine whether Nox2 deficiency influenced the activity and angiogenic potency of miPSC-ECs, WT and Nox2^−/−^ miPSC-ECs were evaluated in a series of *in-vitro* experiments as well as the *in-vivo* Matrigel plug assay. ECs differentiated from Nox2^−/−^ miPSCs (Nox2^−/−^ miPSC-ECs) displayed the expected declines in NADPH oxidase activity and ROS production (Fig. 6A). Measurements of tube formation, cell migration, cell proliferation (Fig. 6B), and acetylated low-density lipoprotein (Ac-LDL) uptake (Fig. 6C) were significantly lower in Nox2^−/−^ miPSC-ECs than in WT miPSC-ECs. Nox2^−/−^ miPSC-ECs were also more sensitive to oxidative stress: measurements of cell survival were significantly lower in Nox2^−/−^ miPSC-ECs than in WT miPSC-ECs when the cells were cultured in H_2_O_2_ concentrations of 400 μM or greater (Fig. 6D), and at 600 μM H_2_O_2_, evidence of cellular senescence (Fig. 6E) and apoptosis (Fig. 6F) was significantly greater in Nox2^−/−^ miPSC-ECs than in WT miPSC-ECs. For the Matrigel plug assay, the cells were suspended in Matrigel and then subcutaneously injected into mice (n = 6 animals per experimental group); seven days later, measurements of both hemoglobin content (Fig. 7A) and capillary density (Fig. 7B) were significantly lower in plugs that contained Nox2^−/−^ miPSC-ECs than in WT miPSC-EC–containing plugs.

### Nox2 deficiency reduces the potency of miPSC-ECs for vascular repair in the ischemic limbs of mice

The effectiveness of WT miPSC-ECs and Nox2^−/−^ miPSC-ECs for improving blood flow after ischemic injury was compared by injecting them into the hind limbs of mice immediately after surgically induced ischemia (n = 8 animals per experimental group). Control assessments were performed in animals that received cell-free injections of saline after injury, blood-flow was monitored before injury and for up to 14 days afterward via laser Doppler perfusion imaging (Fig. 7C, upper panels), and the transplanted cells were identified in histological sections via GFP fluorescence. Compared to measurements in saline-treated animals, blood flow in animals treated with WT miPSC-ECs was significantly greater on Day 3 after injury (and at all subsequent time points) while blood flow in Nox2^−/−^ miPSC-EC–treated animals was not significantly greater until Day 14 (Fig. 7C, lower panel). Blood-flow measurements on Day 14 were also significantly greater in the WT miPSC-EC–treatment group than in Nox2^−/−^ miPSC-EC–treated animals. Dual staining for both GFP and CD31 demonstrated the persistence of both WT miPSC-ECs and Nox2^−/−^ miPSC-ECs in the ischemic limbs (Fig. 7D), and capillary density was significantly higher in the ischemic limbs of either cell-treatment group than in the ischemic limbs of animals treated with saline (Fig. 7E, upper panels). However, arteriole density was no greater in Nox2^−/−^ miPSC-EC–treated animals than in saline-treated animals (Fig. 7E, lower panels), while both capillary density and arteriole density were greater after treatment with WT miPSC-ECs than after Nox2^−/−^ miPSC-EC treatment.

## Discussion

Although excess amounts of ROS are detrimental to cells, when present at an appropriate level, ROS function as signaling molecules that mediate cell growth, migration, and differentiation^32^. For example, the ROS produced by mitochondria may mediate the differentiation of ESCs into cardiomyocytes when glucose levels are high^33^, ESCs require the ROS generated by mechanical strain for differentiation into the cardiovascular lineage^25^, and Nox2-mediated ROS production contributes to the differentiation of cardiac precursor cells into smooth- and cardiac-muscle cells^22^. However, whether Nox2 has a role in the endothelial specification of stem/progenitor cells has yet to be thoroughly investigated. In the present study, we found that the expression of endothelial markers, arterial endothelial markers, and pro-angiogenic cytokines was significantly lower in Nox2^−/−^ miPSC-ECs than in WT miPSC-ECs but increased significantly when Nox2 expression was genetically upregulated or when the cells were treated with H_2_O_2_. Furthermore, our results suggest that the effect of Nox2-produced ROS on the endothelial specification of miPSCs and the angiogenic potency of miPSC-ECs may be mediated by Notch signaling, because the expression of Notch pathway components was also suppressed in Nox2^−/−^ miPSC-ECs and increased in response to Nox2 overexpression or H_2_O_2_ treatment, while arterial-marker expression in both Nox2^−/−^ and WT miPSC-ECs increased in response to Notch1 overexpression. The potential role of Notch signaling in stem-cell activity has been noted previously in reports indicating that the self-renewal of basal stem cells can be stimulated by ROS-mediated Notch-pathway activation^15^, and that Nox1-mediated ROS production may activate Notch1 signaling to control the activity and fate of proliferative progenitor cells in the colon^34^, as well as a considerable amount of evidence from mammalian studies that suggests Notch participates in arterial differentiation^6^,^11^. However, the results presented here are among the first to suggest that Nox2-mediated ROS production and Notch signaling may be linked via a common mechanism (Supplementary Figure 4) that regulates the differentiation of miPSCs into miPSC-ECs, and that this mechanism may be particularly important for the induction of an arterial EC phenotype. The relationship between Nox2 expression and arterial EC specification may also be mediated by VEGF, which was impaired in Nox2^−/−^ miPSC-ECs and has been shown to preferentially promote the expression of arterial markers in ESC-derived ECs^12^.

Nox2 deficiency has been associated with improvements in the blood flow of ischemic limbs when oxidative stress was induced by cigarette smoke^35^ or a high-cholesterol diet^36^, which may seem to contradict our observation that measurements of angiogenic potency were lower for Nox2^−/−^ miPSC-ECs than for WT miPSC-ECs. However, Nox2 deficiency impeded blood-flow recovery in at least two other studies^37^,^38^ as well as in experiments from our own lab (data not shown), that were performed under normal oxidative conditions, and since the detrimental effects associated with excessive levels of ROS are well established, these methodological differences likely explain why our results differ from those obtained when ROS levels are elevated by environmental or dietary factors. Furthermore, Notch signaling can also have varying effects on the vasculogenic activity of stem/progenitor cells and ECs, depending on the stage of development and other factors^39^. For example, Notch induces the differentiation of angioblasts into ECs during early vascular development but regulates the arterial/venous specification of ECs at a later stage^40^, and while VEGF-A activates Notch in ECs, the downstream effects of this interaction include declines in VEGFR2 levels and, consequently, an impaired response to VEGF^41^. Our observations are also consistent with evidence that Notch inhibition impedes tumor angiogenesis^42^,^43^ and that selective Notch pathway activation promotes endothelial regeneration, reduces endothelial apoptosis, and favors angiogenesis^44^,^45^,^46^. Thus, Nox2-mediated ROS production may both promote and inhibit blood vessel formation, depending on the amount of ROS produced by other mechanisms (or the environment)^47^,^48^ and, perhaps, on the pathology of the specific disease being studied.

Because cultured Nox2^−/−^ miPSC-ECs were less resistant to oxidative stress and expressed lower levels of VEGF and angiopoietin-1, the greater perfusion and vascularity observed after treatment with WT miPSC-ECs rather than Nox2^−/−^ miPSC-ECs can likely be explained, at least in part, by differences in the cells’ survival and paracrine activity after transplantation. VEGF and angiopoietin-1 have also been linked to EC specification—VEGF is the first secreted molecule with endothelial specificity during development^49^, and angiopoietin-1 has been shown to promote the differentiation of both ESCs and iPSCs into ECs^50^—which is consistent with the positive correlation observed between the expression of Nox2 and endothelial-lineage marker proteins, as well as the declines in EC-specific cellular functions, such as tube formation and acLDL uptake, associated with Nox2 deficiency. Thus, Nox2 activity may also increase the regenerative potency of transplanted miPSC-ECs by improving the robustness of the EC phenotype.

Though investigations of the role of ROS in the EC specification of stem/progenitor cells are rare^24^,^25^, hypoxia-induced ROS generation promotes arterial EC fate in human pluripotent stem cells^1^, and the ROS produced by Nox activity are known to direct the differentiation of ESCs into cardiomyocytes and smooth-muscle cells^51^,^52^. Furthermore, Lange *et al*.^24^, have shown that the vasculogenic activity induced in embryoid bodies by platelet-derived growth factor BB (PDGF-BB) can be abolished by Nox inhibition, which is consistent with our observations that ephrinB2-positive cells were significantly less common in embryoid bodies that have been differentiated from Nox2^−/−^miPSCs than in embryoid bodies from WT miPSCs. Thus, despite the absence of vascular defects in the embryos of Nox2^−/−^ mice, which can likely be attributed to compensatory mechanisms, our results are consistent with those from several other investigations of the involvement of ROS and/or Nox in vasculogenesis and angiogenesis. We acknowledge that CD31, CD144, VEGF and Ang-1 mRNA levels remained lower in Nox2^−/−^miPSC-ECs than in WT miPSC-ECs after the cells were transfected with Ad-Nox2.Thus, something else than Nox2 may also be responsible for the reduced expression of endothelial markers in Nox2^−/−^miPSC-ECs. Notably, our data also indicate that Nox4 expression gradually increases as miPSCs differentiate into ECs, and Nox4-mediated ROS production has been linked to vasculogenesis and to endothelial and smooth-muscle differentiation in ESCs^53^,^54^, as well as to the differentiation of cardiac precursor cells into cardiac muscle cells^22^,^52^. Thus, Nox4 may also have a role in the endothelial specification of miPSCs, and we will explore this possibility in future studies.

In conclusion, the results presented here are among the first to show that Nox2-mediated ROS production promotes EC specification in differentiating miPSCs by activating the Notch signaling pathway, and that this mechanism is particularly important for the generation of arterial miPSC-ECs. Nox2 expression also appears to contribute to the angiogenic potency of transplanted miPSC-ECs and, consequently, strategies designed to upregulate Nox2 expression may induce a more robust arterial phenotype in miPSC-ECs and improve their effectiveness for regenerative cardiovascular therapy.

## Additional Information

**How to cite this article**: Kang, X. *et al*. Nox2 contributes to the arterial endothelial specification of mouse induced pluripotent stem cells by upregulating Notch signaling. *Sci. Rep.* **6**, 33737; doi: 10.1038/srep33737 (2016).

## Supplementary Material

Supplementary Information
