# Supplementary material for: Nox2 contributes to the arterial endothelial specification of mouse induced pluripotent stem cells by upregulating Notch signaling
Source: Sci Rep. 2016 Sep 19;6:33737. doi: 10.1038/srep33737 (PMC5027389; doi:10.1038/srep33737)

Supplementary Information

Nox2 contributes to the arterial endothelial specification of mouse induced pluripotent stem cells by upregulating Notch signaling

Xueling Kang, Xiangxiang Wei, Xinhong Wang, Li Jiang, Cong Niu, Jianyi Zhang, Sifeng Chen, Dan Meng.

Detailed Methods

Reagents

Lucigenin, NADPH, Drabkin's reagent, SB431542, Accutase, and Alkaline Phosphatase Kit were purchased from Sigma-Aldrich (St. Louis, MO). 2',7'-dichlorodihydrofluorescein diacetate (DCF-DA), dihydroethidium (DHE) and Dil-Ac-LDL were obtained from Invitrogen (Carlsbad, CA). Human vascular endothelial growth factor ($VEGF_{165}$) and bone morphogenetic protein-4 (BMP-4) were obtained from PeproTech Inc. (Rocky Hill, NJ). Matrigel was purchased from BD Pharmingen (San Diego, CA). Antibodies against Oct-4 (sc-5279), VEGF (sc-152), activin receptor-like kinase 1 (ALK1, sc-19546) and GFP (sc-8334) were obtained from Santa Cruz Biotechnology (Santa Cruz, CA). Antibody against stage-specific embryonic antigen 1 (SSEA-1, 4744), Jagged1 (2620), Dll4 (2589), Cleaved Notch1 (NICD1, 2421) and β-actin (4967) were obtained from Cell Signaling (Beverly, MA). Antibodies against CD31 (ab28364), CD144 (ab91064), α-smooth muscle actin (ab5694), Angiopoietin-1 (ab8451), Ephrin B2 (ab150411), Hes1 (ab71559), Flk-1 (ab2349) and HIF-1α (ab16066) were purchased from Abcam (Cambridge, MA). Antibody against Hey1 (AB5714) was purchased from Millipore (Billerica, MA). PE-conjugated CD31 antibody (12-0311) was purchased from eBioscience (San Diego, CA). Murine Notch1 siRNA was obtained from GenePharma (Shanghai, China). The recombinant adenoviral vectors encoding murine Nox2 gene (Ad-GFP-Nox2) and eGFP control (Ad-GFP) were obtained from Cyagen

Biosciences (Guangzhou, China). Notch 1 intracellular domain plasmid (NICD1, #20183) was obtained from Addgene (Cambridge, MA).

Embryoid body formation assay

Mouse iPSCs or mESCs were trypsinized and suspended as single cells. After removal of the medium, the cells were washed 3 times to remove the LIF in the medium. The cells were plated onto 6-well low-attachment plates (Corning Life Sciences) at 1×10^5 cells per well in ESC medium without LIF.

Transfection and viral transduction of miPSCs or miPSC-ECs

miPSCs or miPSC-ECs were transfected with the Notch1 siRNA (100 nmol/L) or the non-targeting negative control siRNA (100 nmol/L), an empty (Control) vector, or vector coding for NICD1 using the Lipofectamine 2000 transfection reagent according to the manufacturer's instructions (Invitrogen, Carlsbad, CA). The transfection medium was replaced after six hours and the cells were incubated for another 48 hours. The recombinant adenoviruses Ad-GFP-Nox2 or Ad-GFP were used to infect miPSCs or miPSC-ECs. For the transduction, a multiplicity of infection (MOI) of 25 was used. No detectable cellular toxicity was observed. Transduction was verified via GFP expression.

Staining

Alkaline phosphatase staining was performed with an Alkaline Phosphatase Kit as directed by the manufacturer's instructions. Immunofluorescent staining was performed as described previously.[1] Briefly, cells were fixed with 4% paraformaldehyde, or tissues from EBs were fixed in ice-cold methanol, permeabilized with 0.1% Triton X-100 in PBS for 10 min, blocked in 5% donkey serum in PBS for 30 min at 37°C, and then incubated with primary antibodies (mouse anti-Oct4, mouse anti-SSEA-1, and mouse anti-EphrinB2). Bound primary antibodies were visualized with fluorescent secondary antibodies, nuclei were counterstained with 4',6-diamidino-2-phenylindole (DAPI; Sigma), and observations were performed with a fluorescence microscope (Leica, Hamburg, Germany).

Teratoma assay

miPSCs were harvested, washed, resuspended in ESC medium (2×10^6 cells/300 μL), and then injected subcutaneously into 6-week-old male NOD/SCID mice. Four weeks later, visible tumors were excised and fixed overnight with 4% paraformaldehyde solution; then, the tumor tissues were paraffin embedded, sectioned, stained with hematoxylin and eosin, and examined by a pathologist.

NADPH oxidase assay

NADPH oxidase (NOX) activity was measured via lucigenin chemiluminescence.[2] Cells were washed five times with ice-cold PBS and centrifuged at 1000 g and 4°C for 10 min; then, the cell-containing pellets were resuspended in lysis buffer (20 mM KH_2PO_4, pH 7.0, 1 mM EGTA, 1 mM phenylmethylsulfonyl fluoride, 10 μg/mL aprotinin, and 0.5 μg/mL leupeptin), and the suspended cells were homogenized with 100 strokes in a Dounce homogenizer on ice. The homogenate (100 μL) was added to 100 μL of phosphate buffer (50 mM, pH 7.0) containing 1 mM EGTA, 150 mM sucrose, 5 μM lucigenin, and 100 μM NADPH, and photon emissions were measured every 15 s for 5 min in a luminometer; sample measurements were blanked by subtracting measurements obtained with the buffer alone before the results were calculated. NOX activity was presented as relative chemiluminescence (light) units per second per milligram of protein. Each experiment was performed three times in three replicate wells.

Measurement of secreted VEGF via ELISA

miPSC-ECs were seeded in a 6-well plate, and the supernatants were collected from the cells 48 hours later. Total cellular protein was measured with a protein assay kit (Bio-Rad Laboratories, Hercules, CA), and VEGF levels were measured with an ELISA assay kit (Neobioscience Technology, Shengzhen, China)

as directed by the manufacturer's instructions. Duplicate assays were performed for each of three independent experiments.

Cell migration assay

Migration was evaluated with transwell chambers (24-well plates, 8-μm pore size, Corning, Inc.) as described previously.[3] Cells (1×10^5) were loaded with ECM2 media into the upper chamber and incubated for 10 hours; then, the non-migrated cells were removed, and the cells that had migrated to the lower compartment were fixed, stained with 0.1% crystal violet, and viewed under a microscope. Cells were counted in five randomly chosen fields per chamber, and three chambers were evaluated for each sample.

Cell proliferation assay

Cell proliferation was evaluated via the incorporation of bromodeoxyuridine (BrdU) into newly synthesized DNA. Cells were seeded into 96-well plates and cultured for the indicated time periods; then, BrdU was added, the cells were cultured for 6 hours, and BrdU incorporation was measured as directed by the manufacture's instructions. (Calbiochem, San Diego, CA). Three replicates were evaluated per sample.

In vitro tube formation assay

The wells of a 48-well plate were filled with 200-μL Matrigel (10 mg/mL, Matrigel Basement Membrane Matrix); then, cell suspensions (1×10^5 cells/well) were added to the Matrigel surface, and the formation of capillary-like structures was evaluated 12-18 hours later. Tube length was measured in five randomly chosen high-power fields in each well under a microscope. Each experiment was performed three times in three replicate wells.

DiI-acetylated low density lipoprotein (DiI-acLDL) uptake

miPSC-ECs were expanded for 7 days, incubated with Dil-acLDL (10 μg/mL, Invitrogen) for 4 hours at 37°C, counterstained with DAPI, washed with PBS, and then viewed and photographed under a fluorescence microscope. The proportion of cells that were positive for Dil-acLDL uptake was quantified in five randomly chosen fields per well, and three wells were evaluated for each sample. Each experiment was performed three times.

Cell viability analysis

Cells were incubated in the indicated concentrations of H_2O_2 for 24 hours; then, the medium was removed and cell viability was evaluated by using a cholecystokinin-8 (CCK-8) assay kit (Dojindo, Tokyo, Japan). Breifly, a solution of cholecystokinin-8 (150 μL) was added to each well; then, the cells were incubated for 2 hours, and CCK-8 absorbance was measured at 450 nm with a microplate reader. Six replicates were evaluated per sample.

Cell apoptosis analysis

Cells were incubated in H_2O_2 (600 μM) for 24 hours, harvested, fixed in 70% ethanol for 1 hour at 4 °C, and then stained with propidium iodide and annexin V-fluorescein isothiocyanate for 15 min at 37 °C. Measurements were performed on a flow cytometer; three replicates were evaluated for each sample.

Senescence assay

Cells were added to a 12-well plate, cultured until ~70-80% confluent, incubated in H_2O_2 (600 μM) for 6 hours, fixed in 2% paraformaldehyde, and stained for β-galactosidase expression as directed by the manufacturer's instructions (BioVision, Milpitas, CA); then, the senescent (i.e., blue-stained) cells were quantified via inverted microscopy in five randomly selected regions per well, three wells per sample.

References

1. Wu, Y. et al. Oxidative stress inhibits adhesion and transendothelial migration, and induces apoptosis and senescence of induced pluripotent stem cells. *Clin Exp Pharmacol Physiol* **40**, 626-34 (2013).
2. Meng, D., Lv, D.D. & Fang, J. Insulin-like growth factor-I induces reactive oxygen species production and cell migration through Nox4 and Rac1 in vascular smooth muscle cells. *Cardiovasc Res* **80**, 299-308 (2008).
3. Meng, D. et al. Arsenic promotes angiogenesis in vitro via a heme oxygenase-1-dependent mechanism. *Toxicol Appl Pharmacol* **244**, 291-9 (2010).

Supplementary Table 1. Primers used for qRT-PCR

Gene	Sequence (5′-3′)	
Nox1	Sense	5'-ATTGCCACTGTAGCTTTG-3'
	Antisense	5'-CCTTATGGTCATCCCACT-3'
Nox2	Sense	5'-GGTGATGTTAGTGGGAGC-3'
	Antisense	5'-AGGAAGTTGGCATTGTTC-3'
Nox3	Sense	5'-CACGAGTTATTCTGGGTTC-3'
	Antisense	5'-AAGTTCCGACTGACAGGTA-3'
Nox4	Sense	5'-AAACACCTCTGCCTGCTCAT-3'
	Antisense	5'-CGCCCAACATTTGGTGAATG-3'
p22phox	Sense	5'-CCCTCCACTTCCTGTTGTC-3'
	Antisense	5'-CCCTCACTCGGCTTCTTT-3'
p47phox	Sense	5'-GATGAAGACAAAGCGAGGTT-3'
	Antisense	5'-CAGATACATGGATGGGAAATAG-3'
p67phox	Sense	5'-CAACATAGGCTGCGTGAA-3'
	Antisense	5'-TCTTTGATAGCAAGGTCGT-3'
CD31	Sense	5'-AGAGGACCAGTCCAAGGGTT-3'
	Antisense	5'-ATGGGCTAATCCAGAGTCAGT-3'
CD144	Sense	5'-CACGGACAAGATCAGCTCCT-3'
	Antisense	5'-CACATAGTGGGGCAGCGATT-3'
eNOS	Sense	5'-GCATGGGCAACTTGAAGAGTG-3'
	Antisense	5'-TACAGGGCCCATCCTGCTGA-3'
vWF	Sense	5'-AAGTTGGGCTAGTCTCTGCG- 3'
	Antisense	5'-GGGAGCAGAGGGATGTCTCG-3'
VEGF	Sense	5'-GAGCTCATGGACGGGTGAG-3'
	Antisense	5'-CTGGGACCACTTGGCATGG-3'
KDR	Sense	5'-ACACGGTCATCCTCACCAAC-3'
	Antisense	5'-TTGTTTGGCCGGGTCTGTAG-3'
Angiopoietin-1	Sense	5'-AACAGGAGGTTGGTGGTTCG-3'
	Antisense	5'-GGTGACATGACATAACCACTTGC-3'
Angiopoietin-2	Sense	5'-CAGCAGCATGACCTAATGGA-3'
	Antisense	5'-ACAGTCTCTGAAGGTGGTTTG-3'
Tie2	Sense	5'-GAAGGGCAAGATGGATAGGGCTCGC-3'
	Antisense	5'-ATCCTTCTGGTCCACTACACCTTTC-3'
PDGF-BB	Sense	5'-CTGAGCGACCACTCCATCC-3'
	Antisense	5'-GCACTCGGCGATTACAGC-3'
FGF-2	Sense	5'-GGCTGCTGGCTTCTAAGTGT-3'
	Antisense	5'-GTCCCGTTTTGGATCCGAGT-3'
Ephrin B2	Sense	5'-GTGTGCCAGACAAGAGCCAT-3'
	Antisense	5'-TGCTAGAACCTGGATTTGGCTT-3'
ALK1	Sense	5'-CTTGGGGAGCTTCAGAAGGGG-3'
	Antisense	5'-GGTGGCCTCCAGCATCAGAGA-3'
Nrp1	Sense	5'-GCTGTGAAGTGGAAGCACCT-3'
	Antisense	5'-GGAAGTCATCACCTGTGCCA-3'
Notch1	Sense	5'-TGTTGTGCTCCTGAAGAACG-3'
	Antisense	5'-TCCATGTGATCCGTGATGTC-3'
Dll4	Sense	5'-TGCCTGGGAAGTATCCTCAC-3'
	Antisense	5'-GTGGCAATCACACACTCGTT-3'
Jag1	Sense	5'-ATACACGTGGCCATCTCTGC-3'

	Antisense	5'-CCGCTTCCTTACACACCAGT-3'
Jag2	Sense	5'-TGTGCGCTCGACATTGATGA-3'
	Antisense	5'-CCGGGAGGCAATCACAGTAA-3'
Hes1	Sense	5'-CTACCCCAGCCAGTGTCAAC-3'
	Antisense	5'-AAGCGGGTCACCTCGTTCAT-3'
Hey1	Sense	5'-CGTGGGAAAGGGATGGTTGA-3'
	Antisense	5'-CAAGTTTCCATTCTCGTCCGC-3'
Hey2	Sense	5'-GGCTACAGGGGGTAAAGGCTA-3'
	Antisense	5'-AGATGAGAGACAAGGCGCAC-3'
Oct4	Sense	5'-TCACTCACATCGCCAATCA-3'
	Antisense	5'-GGTGTCCCTGTAGCCTCATA-3'
Nanog	Sense	5'-ATTCTGGGAACGCCTCAT-3'
	Antisense	5'-GCTTTTGTTTGGGACTGGTA-3'

SUPPLEMENTARY FIGURE LEGENDS

Supplementary Figure 1. The mRNA expression profile of NOX subunits in miPSCs and Nox2 deficiency reduces NADPH oxidae activity and ROS production in miPSCs.

A, Quantitative PCR analyses show that Nox2 and Nox4 were highly expressed, and Nox1 was expressed at low levels in WT miPSCs (n=3; ***P*<0.01 vs Nox1). **B**, Quantitative PCR analyses showed that Nox2 expression was knocked out, and that the mRNA levels of Nox1, Nox4, p22phox, p47phox, and p67phox between Nox2$^{-/-}$ miPSCs and WT miPSCs were similar. WT miPSCs and Nox2$^{-/-}$ miPSCs were assessed for NADPH oxidase activity (**C**) and ROS production (**D** and **E**), showing the decrease of ROS levels and NADPH oxidase activity in Nox2$^{-/-}$ miPSCs. (n=3; **P*<0.05, ***P*<0.01 vs WT miPSCs).

Supplementary Figure 2. Nox2 and Nox4 expression gradually increases in miPSCs and mESCs during the differentiation of stem cells into endothelial cells.

A and **B**, mRNA levels of Nox2 and Nox4 were measured by quantitative real-time RT-PCR at day 0, 3, 5, 7 of EC differentiation in miPSCs and mouse embryonic stem cells (mESCs). (n=3; **P*<0.05, ***P*<0.01 vs day 0). **C**, Quantitative PCR analyses show that the mRNA levels of Nox2 and Nox4 were significantly upregulated in WT miPSC-ECs at day 14 of differentiation compared with WT miPSCs (n=3; ***P*<0.01 vs WT miPSCs).

Supplementary Figure 3. Nox2 deficiency reduces Flk-1 positive cells and the expression of HIF-1α and VEGF in differentiating miPSCs, but does not affect Oct4 and Nanog expression during EB differentiation.

A, Flow cytometry analysis of Flk-1 positive cells derived from WT miPSCs and Nox2$^{-/-}$ miPSCs at day 5 of EC differentiation (n=3; **P*<0.05 vs WT miPSCs). **B** and **C**, mRNA levels of Oct4 and Nanog were measured by quantitative real-time RT-PCR during spontaneous differentiation in EB model as indicated time points in WT and Nox2$^{-/-}$ miPSCs (n=3). **D**, mRNA levels of PDGF-BB, FGF-2 and Ang-2 were measured by quantitative real-time RT-PCR in WT and Nox2$^{-/-}$ miPSC-ECs. **E**, Protein levels of HIF1α

were evaluated by immunobloting in WT and Nox2$^{-/-}$ miPSC-ECs that had been cultured in a hypoxic chamber (Hypoxia) for 12 hours. **F**, Protein levels of VEGF in the supernatant of WT miPSC-ECs and Nox2$^{-/-}$ miPSC-ECs were evaluated via ELISA. (n=3; **P*<0.05, vs WT miPSC-ECs).

Supplementary Figure 4. Schematic representation of Nox2-derived ROS regulating miPSC differentiation into arterial endothelial lineage.

Supplementary Figure 1

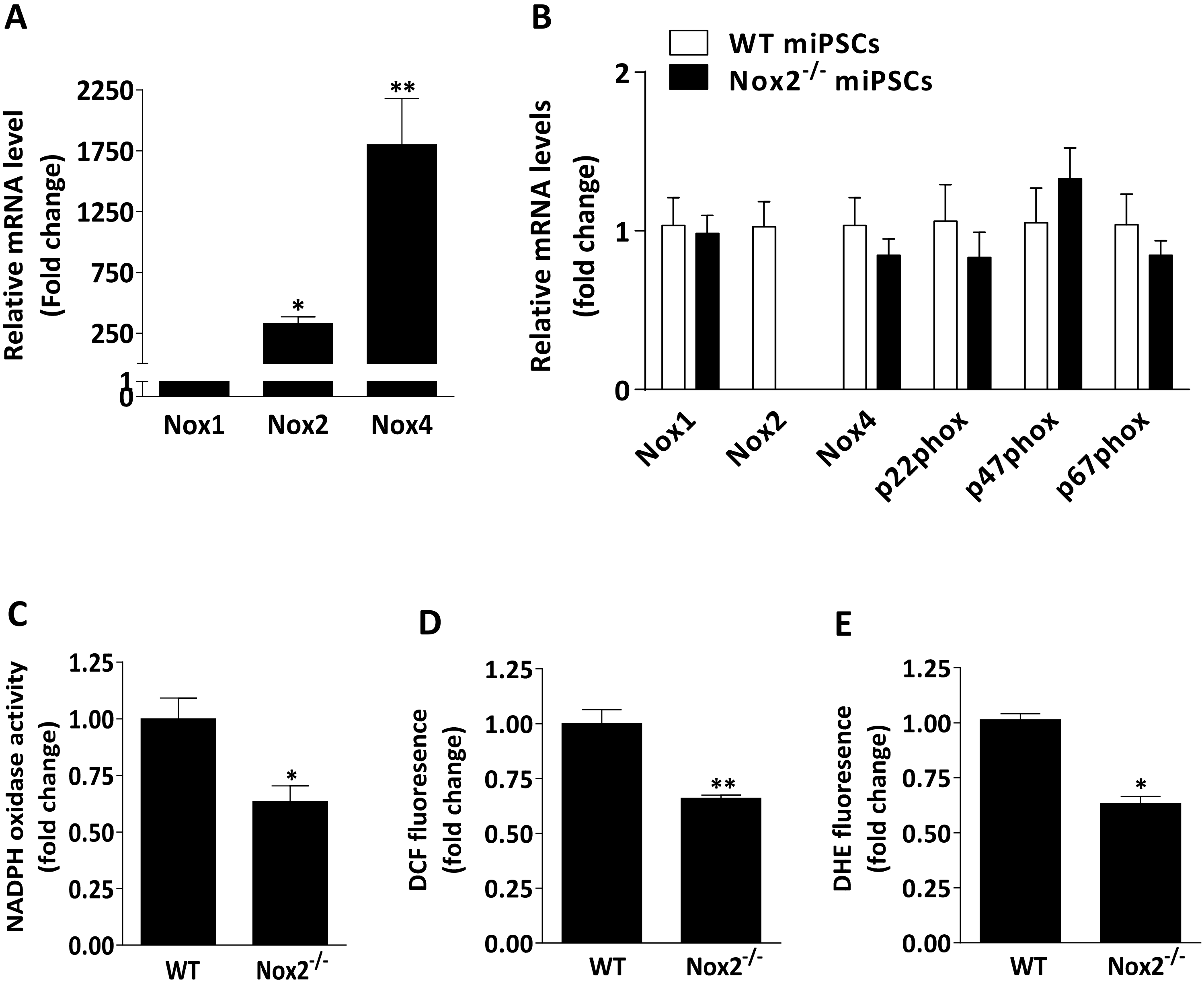

Supplementary Figure 2

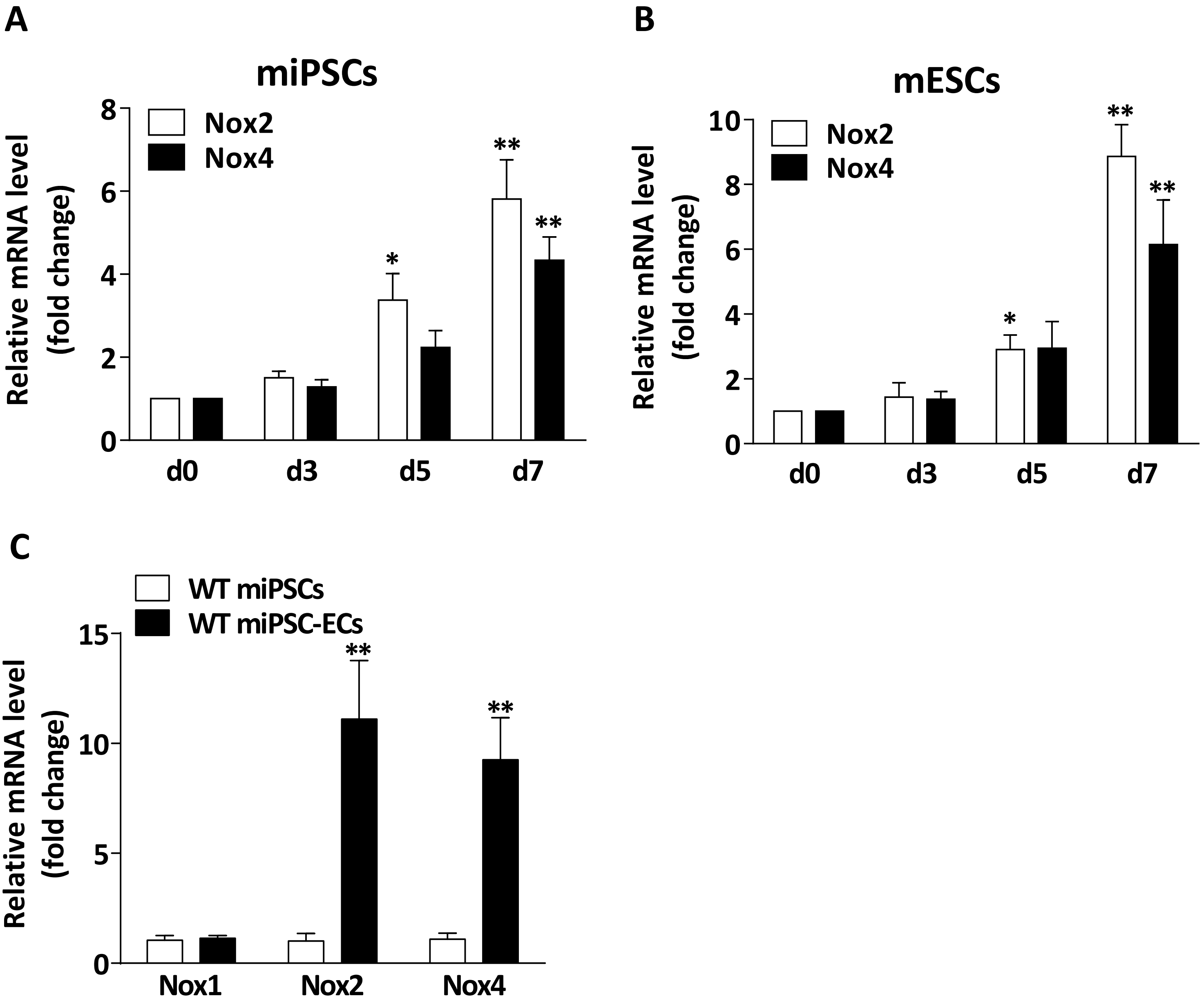

Supplementary Figure 3

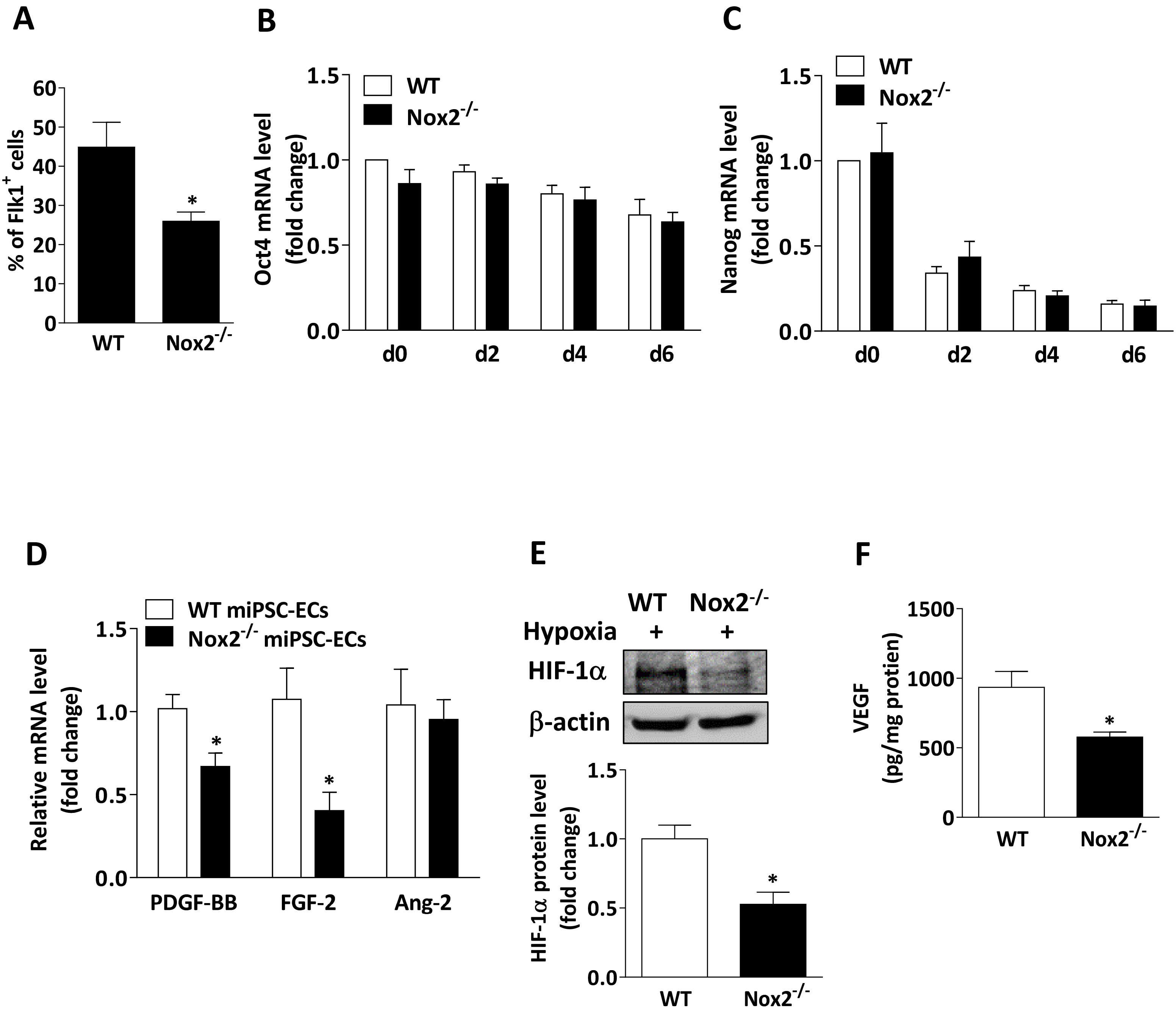

Supplementary Figure 4

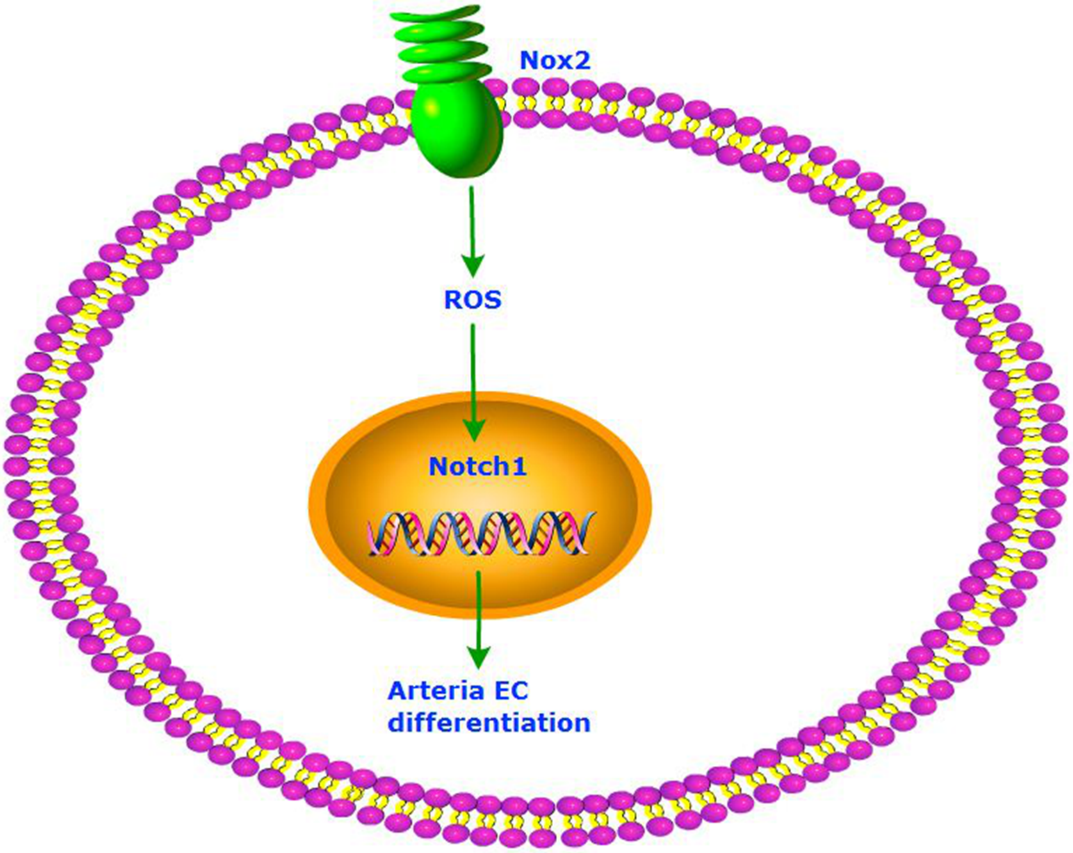

Supplement: Supplementary Information [file srep33737-s1.pdf]
